# Klinefelter Syndrome and the Need for a Multi-Disciplinary Approach

**DOI:** 10.3389/frph.2021.622144

**Published:** 2021-04-22

**Authors:** Andreas Espehana, Charlotte Tomlinson

**Affiliations:** ^1^GKT School of Medical Education, King's College London, London, United Kingdom; ^2^Clinical Genetics & Centre for PGD, Guy's Hospital, London, United Kingdom

**Keywords:** urology, Klinefelter syndrome, infertility, multi-disciplinary, hypogonadism, genetics

## Introduction

Klinefelter's syndrome (KS) occurs as a *de novo* event in 1 in 600 males. While 80% of cases of KS have the 47 XXY Karyotype, it is known that some patients may have more than one extra X chromosome or express mosaicism 46XY/47XXY (1). It is the most frequently occurring chromosome abnormality in infertile men. Besides infertility, this condition also affects the mental and physical health of these men.

## Problems Faced in Patients With Ks

Men with KS often suffer from hypergonadotropic hypogonadism due to improper development of their testes. Low testosterone results in a range of physical and psychological symptoms that spans over seven different specialities of medicine.

The gradual depletion of their spermatogonial stem cells in the testes during puberty means that by the time they reach adulthood, they may be severely oligospermic or even azoospermic (2), thus, severely affecting their fertility.

The low levels of testosterone can lead to the absence or regression of secondary sex characteristics during puberty, leading to reduced facial and body hair, small testes and gynecomastia.

Furthermore, they may also develop anemia, muscle wasting, reduced bone mass or density, abdominal adiposity, dyslipidemia, and hypertension. Studies have demonstrated that these have a higher risk of developing metabolic syndrome, type 2 diabetes mellitus, osteoporosis, venous thromboembolism and autoimmune diseases (3) such as rheumatoid arthritis and systemic lupus erythematosus.

While most men have normal intellectual development, some may suffer from a delay in speech and language development, particularly in their expression of emotion.

In terms of sexual function, they may experience erectile dysfunction, reduced libido and difficulty in obtaining an orgasm. Thus, affecting their quality of life and personal relationships as well.

## Management of Ks

Due to the wide range of problems men with KS face, the management requires input from a multi-disciplinary team (MDT), all of whom are well-versed with this condition. The seven different specialities we recommend are urology, endocrinology, fertility, radiology, embryology, psychosexual, and genetic counseling. Genetic counseling helps the patient and their partner understand what has caused their condition and what the risk for biological children and alternative options for having a family. It helps them to consider how they manage the emotional changes they may feel around their diagnosis and both their and their partners concerns for the future. Psychosexual counseling helps those men with KS who may have difficulties with sex including erectile dysfunction and delayed to absent ejaculation. Sexual issues that have a significant impact on relationships but people are understandably reluctant to raise these issues and so this gives KS men and their partners the opportunity to share concerns and get the help they need for a more fulfilling relationship.

Endocrinologist can manage and treat the physical symptoms brought about by the low levels of testosterone, using testosterone replacement therapy (TRT). Urologist can offer fertility treatment such as Hormone Manipulation Therapy (HRT) to increase their sperm count and microsurgical sperm extraction. Together with the fertility specialist and embryologist, can assist men in conceiving a child with their partner through *In-vitro* fertilization. However, the exogenous testosterone from TRT can inhibit sperm production, in these patients, leading to difficulty in conception with their partner. This example illustrates the difficulties in the management of KS patients, as their needs spill over into other disciplines of medicine.

As these patients are predisposed to developing secondary diseases mentioned earlier, they require long term monitoring of their blood pressure, glucose controls, bone health, and lipid levels.

Patients differ greatly in terms of the symptoms they experience, and some may reach adulthood without noticing these symptoms. Some may even develop symptoms but have their underlying chromosomal abnormality go undiagnosed. Hence, many of the patients that come to our clinic are already in adulthood, as they have only been diagnosed with KS after being investigated for infertility.

## Multi-Disciplinary Team Approach

As the patient's condition and needs are complex, they often extend beyond the scope of each speciality. An MDT approach allows the clinicians managing the different aspects of the patients' needs to collaborate effectively and efficiently, allowing for a more comprehensive and holistic approach to patient care. The MDT consists of specialist from the seven mentioned areas of medicine, along with a specialist nurse practitioner, pharmacist, administrative staff, and a patient liaison from the KS Association.

Having the seven different specialities in our one clinic enables the team to come up with a single cohesive management plan for each patient. Patients will only see the specialist that they require to meet their specific needs, all in the same day. This greatly increases healthcare efficiencies as the waiting time required for patients to see all the necessary specialists are greatly reduced. Often patients wait over a year in primary care before they are seen by a single one of these specialists and subsequently wait the equivalent times to see the next.

Currently, the MDT clinic is being conducted at Guys & St.Thomas' Hospital, a tertiary teaching hospital in London. Before each patient comes to the clinic, they are discussed within the team to ensure that everyone has a general idea of the patient's background, the problems they face, which specialties they need to consult with, and any potential issues that may arise from treatment. After the clinic ends, another team discussion is held for each patient to ensure a consistent management plan. This communication and collaboration within the team is the key to how this clinic can potentially improve the outcomes for these men.

However, running the MDT clinic comes with a set of challenges. Firstly, having a large number of clinicians involved means that coordinating a time and place for the clinic to take place can be challenging. The frequency of the clinic is limited to the availability of all our clinicians, hence the clinic is only run once every 5 weeks. Secondly, the irregularity of this clinic means that as awareness rises, the number of referrals will to and thus waiting times may increase as well. Lastly, as we receive referrals from across the country, some patients may have to travel a significant distance to attend our clinic in central London.

The clinic aims to increase awareness and education of KS in the healthcare community. One of the main reasons for the long waiting time for patients in primary care could be attributed to the lack of awareness of the possibility of KS as a diagnosis for these patients. Through education, we hope that more men with KS can be diagnosed earlier and spend less time in primary care before they see a specialist in their condition.

## Author Contributions

AE: wrote the manuscript. CT: co-wrote the manuscript. All authors contributed to the article and approved the submitted version.

## Conflict of Interest

The authors declare that the research was conducted in the absence of any commercial or financial relationships that could be construed as a potential conflict of interest.

## References

[1] Radicioni AF Ferlin A Balercia G Pasquali D Vignozzi L Maggi M. Consensus statement on diagnosis and clinical management of Klinefelter syndrome. J Endocrinol Invest. (2010) 33:839–50. 10.1007/BF03350351 21293172

[2] Gies I De Schepper J Goossens E Van Saen D Pennings G Tournaye H. Spermatogonial stem cell preservation in boys with Klinefelter syndrome: to bank or not to bank, that's the question. Fertil Steril. (2012) 98:284–9. 10.1016/j.fertnstert.2012.04.023 22608314

[3] Salzano A Arcopinto M Marra AM Bobbio E Esposito D Accardo G. Management of endocrine disease: Klinefelter syndrome, cardiovascular system, and thromboembolic disease: review of literature and clinical perspectives. Eur J Endocrinol. (2016) 175:R27–40. 10.1530/EJE-15-1025 26850445

